# Depression and associated factors in older adults in South Africa

**DOI:** 10.3402/gha.v6i0.18871

**Published:** 2013-01-18

**Authors:** Karl Peltzer, Nancy Phaswana-Mafuya

**Affiliations:** 1 HIV/AIDS/SIT/and TB (HAST), Human Sciences Research Council, Pretoria, South Africa; 2 Department of Psychology, University of Limpopo, Turfloop, South Africa; 3 Office of the Vice Chancellor, Nelson Mandela Metropolitan University, Port Elizabeth, South Africa

**Keywords:** self-reported depression symptoms, risk factors, older adults, South Africa, WHO SAGE

## Abstract

**Background and objective:**

Late-life depression is an important public health problem because of its devastating consequences. The study aims to investigate the prevalence and associated factors of self-reported symptom-based depression in a national sample of older South Africans who participated in the Study of Global Ageing and Adult Health (SAGE wave 1) in 2008.

**Methods:**

We conducted a national population-based cross-sectional study with a probability sample of 3,840 individuals aged 50 years or above in South Africa in 2008. The questionnaire included socio-demographic characteristics, health variables, anthropometric and blood pressure measurements as well as questions on depression symptoms in the past 12 months. Multivariable regression analysis was performed to assess the association of socio-demographic factors, health variables, and depression.

**Results:**

The overall prevalence of symptom-based depression in the past 12 months was 4.0%. In multivariable analysis, functional disability, lack of quality of life, and chronic conditions (angina, asthma, arthritis, and nocturnal sleep problems) were associated with self-reported depression symptoms in the past 12 months.

**Conclusions:**

Self-reported depression in older South Africans seems to be a public health problem calling for appropriate interventions to reduce occurrence. Factors identified to be associated with depression, including functional disability, lack of quality of life, and chronic conditions (angina, asthma, arthritis, and nocturnal sleep problems), can be used to guide interventions. The identified protective and risk factors can help in formulating public health care policies to improve quality of life among older adults.

Late-life depression is an important public health problem because of its devastating consequences (1). It is associated with an increased risk of morbidity and decreased physical, cognitive, and social functioning (2). Major depression defined by the *Diagnostic and Statistical Manual of Mental Disorders*, third edition (DSM-III) (3), has been found to be less prevalent among older adults living in communities than among younger community residents (4). The prevalence of major depressive disorders at any given time in community samples of adults aged 65 and above ranges from 1 to 12.3% (1, 5–8), and clinically significant depressive symptoms are present in 9.8–39% of community-dwelling older adults (2, 9–13). There have been no studies investigating depression in older adults in South Africa; only one study in the general population found a prevalence of major depression of 9.7% for lifetime and 4.9% for the 12 months prior to the interview (14).

Various factors have been identified to be associated with depression in older adults, including:

1. Socio-economic (female gender, race or ethnicity, widowhood, separated/divorced marital status, poor economic status, rural residence) (7–10, 15–17);
2. Social (stressful life events such as bereavement, including socio-economic stress factors) (12, 18, 19);
3. Unhealthy behaviour [poor dietary habits (20); lack of physical activity (21, 22); and risk factors, namely hypertension (12) and obesity (23, 24)];
4. Disease and chronic conditions, including stroke (9, 25), cardiovascular disease (12, 17, 26), type II diabetes (27), arthritis (9), asthma (28), sleep problems (29–31);
5. Cognitive impairment (9, 12, 32, 33);
6. Functional disability (8, 10, 12); and
7. Low quality of life (8, 11, 34).

The study aims to investigate the prevalence and associated factors of self-reported symptom-based depression in a national sample of older South Africans who participated in the Study of Global Ageing and Adults Health (SAGE wave 1) in 2008.

## Methods

### Sample and procedure

We conducted a national population-based cross-sectional study with a probability sample of 3,840 individuals aged 50 years or above in South Africa from January to September in 2008. The SAGE sample design entails a two-stage probability sample that yields national and sub-national estimates to an acceptable precision at provincial level, by locality type (urban and rural) and population group (including black, coloured, Indian or Asian, and white). The individual response rate among those aged 50 years or older was 77%. The Global Study on Ageing (SAGE wave 1) survey was carried out in South Africa in partnership among the World Health Organization (WHO), the National Department of Health, and the Human Sciences Research Council (HSRC). The study was approved by the HSRC Research Ethics Committee and the National Department of Health.

### Measures

The questionnaire used for this study is the individual questionnaire from SAGE, found at the study website http://www.who.int/healthinfo/systems/sage/en/.

### Depression

Symptom-based depression in the past 12 months was assessed based on the World Mental Health Survey version of the Composite International Diagnostic Interview (35). The diagnosis of depression was based on the International Classification of Diseases, 10th revision (ICD-10), diagnostic criteria for research (DCR) for depressive episodes (36) and was derived from an algorithm that took into account respondents reporting symptoms of depression during the past 12 months (37). Participants endorsed at least 4 of 10 depressive symptoms lasting 2 weeks most of the day or all of the day. According to the ICD–10–DCR criterion B, at least two of the following three symptoms needed to be present: depressed mood, loss of interest, and fatigability. In addition, the ones who responded affirmatively to the question, ‘Have you been taking any medications or other treatment such as attending therapy or counselling sessions for depression during the last 12 months?’ was added to the symptom-based depression.

Nocturnal sleep problems were assessed with the question, ‘Overall in the last 30 days, how much of a problem did you have with sleeping, such as falling asleep, waking up frequently during the night, or waking up too early in the morning?’ Response options were none, mild, moderate, severe, and extreme. Responses were collapsed in two categories: none/mild/moderate and severe/extreme (31).

### Fruit and vegetable consumption

Fruit and vegetable consumption was assessed with the questions ‘How many servings of fruit do you eat on a typical day?’ and ‘How many servings of vegetables do you eat on a typical day?’ Researchers were trained to show all respondents a nutrition risk factor card that indicates both in writing and in pictures general categories, amounts, and examples of fruits and vegetables in an attempt to standardise the serving size and number of servings reported (38). Insufficient fruits and vegetable consumption were defined as less than five servings of fruits and/or vegetables a day (39).

### Blood pressure (systolic and diastolic)

Blood pressure was measured three times on the right arm/wrist of the seated respondent using an automated recording device (OMRON R6 Wrist Blood Pressure Monitor, HEM-6000-E, Omron Healthcare Europe, B.V., Hoofddorp, The Netherlands).

Out of three measurements, the average of the last two readings was used. In accordance with the Seventh Report of the Joint National Committee on Prevention, Detection, Evaluation, and Treatment of High Blood Pressure, individuals with systolic blood pressure ≥140 mmHg and/or diastolic blood pressure ≥90 mmHg and/or who reported the current use of antihypertensive medication were considered to be suffering from high blood pressure (40).

### Tobacco use

Lifetime tobacco used was assessed with the question ‘Have you ever smoked tobacco or used smokeless tobacco?’ Lifetime tobacco users were asked ‘Do you currently use (smoke, sniff, or chew) any tobacco products such as cigarettes, cigars, pipes, chewing tobacco, or snuff?’ The response options were ‘Yes, daily’, ‘Yes, but not daily’, and ‘No, Not at all’. These questions are based on the WHO Guidelines for Controlling and Monitoring the Tobacco Epidemic (41).

### Alcohol use

Lifetime alcohol use was assessed with the question ‘Have you ever consumed a drink that contains alcohol (such as beer, wine, spirits, etc.)?’ Response options were ‘Yes’ or ‘No, never’. Lifetime alcohol users were asked about current (past month) alcohol use, and current alcohol users were asked ‘During the past 7 days, how many drinks of any alcoholic beverage did you have each day?’ (42).

### Height and weight

Height and weight were measured, and body mass index (BMI) was used as an indicator of obesity (≥30 kg/m^2^). BMI was calculated as weight in kilogram divided by height in metre squared. Obesity was defined as ≥30 BMI and underweight <18.5 BMI.

### Physical activity

Physical activity was measured using the General Physical Activity Questionnaire (GPAQ) version 2. The instrument gathered information on physical activity in three domains (activity at work, travel to and from places, and recreational activities), as well as time spent on sitting. The questionnaire also assessed vigorous and moderate activities performed at work and on recreational activities.

Information on the number of days in a week spent on different activities and time spent in a typical day for each activity was also recorded (43). For physical activity, in addition to the total minutes of activity, the activity volume was also computed by weighing each type of activity by its energy requirement in metabolic equivalents (METs). One MET was defined as the energy cost of sitting quietly and was equivalent to a calorie consumption of 1 kcal/kg/hour. A MET-minute showed the total activity volume on a weekly basis, calculated by multiplying the time spent on each activity during a week by the MET-values of each level of activity. MET-values for different level of activities were set as 4 MET for moderate intensity physical activity, 8 MET for vigorous physical activity, and 4 MET for transport-related walking or cycling. The total physical activity for GPAQ2 was calculated as the sum of total moderate, vigorous, and transport-related activities per week.

The number of days and total physical activity MET minutes per week were used to classify respondents into three categories of low, moderate, and high level of physical activity.

1. High physical activity. A person reaching any of the following criteria is classified in this category: vigorous-intensity activity on at least 3 days achieving a minimum of at least 1,500 MET-minutes per week or 7 or more days of any combination of walking, moderate or vigorous intensity activity achieving a minimum of at least 3,000 MET-minutes per week.
2. Moderate physical activity. A person not meeting the criteria for the ‘high’ category but meeting any of the following criteria is classified in this category: 3 or more days of vigorous-intensity activity of at least 20 min per day, 5 or more days of moderate-intensity activity, or walking of at least 30 min per day OR 5 or more days of any combination of walking and moderate or vigorous intensity activity achieving a minimum of at least 600 MET-minutes per week.
3. Low physical activity. A person not meeting any of the aforementioned criteria falls in this category. Physical inactivity was defined as those who had low levels of physical activity; moderate and high levels of physical activity were collapsed in further analysis (43).

### Functional ability

Functional ability was measured by the 12-item WHO Disability Assessment Schedule, version 2 (WHODAS-II) (44), designed to measure disability from responses to questions on physical functioning in a range of activities of daily life as well as instrumental activities of daily life. Participants were asked about difficulties in the past 30 days with performing activities of daily living, such as standing, taking care of household responsibilities, learning a new task as well as instrumental activities of daily living, such as getting dressed and participation in community activities. Responses to these questions were scored using a 5-point Likert-type response scale, ‘none’, ‘mild’, ‘moderate’, ‘severe’, and ‘extreme/cannot do’. The scores assigned to each of the items – ‘none’ (1), ‘mild’ (2), ‘moderate’ (3), ‘severe’ (4), and ‘extreme’ (5) – were summed. This method is referred to as simple scoring because the scores from each of the items are simply added up without recoding or collapsing of response categories; thus, there was no weighting of individual items. These responses were used to create a score of overall disability (44). The computed WHODAS score ranged from 0 to 36 and was later transformed into 0–100 with 100 being severe/extreme disability (44).

### Social cohesion

Social cohesion was measured with nine items, starting with the introduction ‘How often in the last 12 months have you…’ e.g. attended any group, club, society, union, or organisational meeting?’ Response options ranged from never = 1 to daily = 5. The scores assigned to each of the items – ‘never’ (1), ‘once or twice a year’ (2), ‘once or twice per month’ (3), ‘once or twice per week’ (4), and ‘daily’ (5) – were summed. These responses were used to create a score of overall social cohesion. Cronbach's alpha for this social cohesion index in this sample was 0.73.

### Quality of life

Quality of life was assessed with the World Health Organization Quality of Life (WHOQOL)-8 containing eight items that were empirically derived from the WHOQOL-Bref (45). The summative model was used to produce an index. Cronbach's alpha for the WHOQOl-8 was 0.85 in this sample.

### Economic or wealth status

To estimate economic or wealth status, a random-effects probit model was used to identify indicator-specific thresholds that represent the point on the wealth scale above which a household is more likely to own a particular asset than not. This enabled an estimation of an asset ladder. These estimates of thresholds, combined with actual assets observed to be owned for any given household, were used to produce an estimate of household-level wealth status. This was used to create wealth Quintiles (46).

### Chronic conditions

Other chronic conditions, such as stroke, angina, diabetes, arthritis, and asthma, were assessed by self-report.

### Cognitive impairment

Cognitive impairment was defined as those who endorsed the question of having severe or extreme difficulty with concentrating or remembering things in the past 30 days. Cognitive tests included verbal learning (a score of 4 or lower represented impairment in verbal learning) (47); verbal recall (a score of 4 or lower represented impairment in verbal recall) (47), and word fluency (a score of 8 or lower represented impairment in word fluency) (47).

### Data analysis

The data were entered using CSPro and analysed using STATA Version 10. The data were weighted using post-stratified individual probability weights based on the selection probability at each stage of selection. Individual weights were post-stratified by province, sex, and age groups according to the 2009 Medium Mid Year population estimates from Statistics South Africa (Available at: http://www.statssa.gov.za/publications/P0302/P03022009.pdf). Weights were not normalised. Chi-square tests of significance were used to compare distribution of socio-demographic and health variables by depression prevalence. Associations between the key outcome of depression and socio-demographic, social, and health variables were evaluated calculating odds ratios (OR). Unconditional multivariable logistic regression was used for the evaluation of the impact of explanatory variables for the outcome of depression (binary dependent variable). All variables statistically significant at the *p*<0.05 levels in bivariate analyses were included in the multivariable models. In the analysis, weighted percentages are reported. Both the reported 95% confidence intervals and the *p*-value are adjusted for the multi-stage stratified cluster sample design of the study.

## Results

### Characteristics of study participants

The total sample included 3,840 South Africans aged 50 years and above, 44.1% men and 55.9% women. The most prevalent population group was African Black (74%), almost half (49.9%) were between 50 and 59 years old. The educational level of most participants (71.6%) was lower than secondary school education and almost two-thirds (64.9%) lived in an urban area. Almost half (46.7%) of older adults were obese and 77.3% had hypertension, 20.4% were daily tobacco users, and 9.2% had diabetes. In addition, 4.0% had had a stroke, 5.2% angina, 4.9% asthma, 24.7% arthritis, and 8.9% a nocturnal sleep problem. More than half (60.5%) engaged in low physical activity, 20.4% were daily tobacco users, a small proportion (3.7%) were hazardous or harmful alcohol users, and 67.7% had insufficient fruit and vegetable intake. The mean functional disability was 20.8 (SD = 20.1, range: 0–97.2) and the mean of quality of life was 47.1 (SD = 12.5, range: 0 = 80.0). The overall prevalence of past 12 months depression was 4.0% (see Table 1). The mean functional disability was 33.2 (SD = 20.2; range: 0–91.7) among those with depression and 20.3 (SD = 20.0, range: 0–97.2) compared to those without depression.

**Table 1:** Sample characteristics and prevalence of depression among older South Africans

Variables	Total sample	Depression in past 12 months	p*
Sociodemographics	*N* (%)	*N* (%)	
All	3,840	160 (4.0)	
Age	1,695 (49.9)	95 (4.5)	0.61
50–59	1,233 (30.6)	40 (3.8)	
60–69	661 (14.0)	21 (3.2)	
70–79	251 (5.5)	4 (1.9)	
80 and over			
Gender			
Male	1,638 (44.1)	63 (3.8)	0.42
Female	2,202 (55.9)	97 (4.1)	
Population group			
African Black	2,053 (74.0)	71 (4.2)	0.90
White	269 (9.3)	14 (3.1)	
Coloured	655 (12.8)	31 (2.7)	
Indian or Asian	307 (3.8)	21 (4.3)	
Marital status			
Single	512 (14.3)	15 (3.7)	0.12
Married	2,007 (55.9)	78 (4.1)	
Separated/divorced	230 (5.9)	14 (5.1)	
Widow	1,020 (23.9)	50 (3.6)	
Educational level			
No schooling	854 (25.2)	49 (3.3)	0.20
Less than primary	803 (24.0)	37 (4.5)	
Primary	779 (22.4)	38 (4.6)	
Secondary	923 (28.3)	36 (4.0)	
Wealth			
Low	1,482 (40.6)	39 (3.6)	0.64
Medium	731 (18.2)	38 (3.8)	
High	1,608 (41.2)	83 (4.4)	
Geolocality			
Rural	1,276 (35.1)	44 (4.5)	0.64
Urban	2,561 (64.9)	116 (3.7)	
Health variables			
Chronic conditions			
High blood pressure	2,842 (77.3)	118 (3.8)	0.91
Stroke	139 (4.0)	14 (11.5)	0.01
Angina	219 (5.2)	31 (16.4)	<0.001
Diabetes	360 (9.2)	25 (6.0)	0.23
Obesity (BMI ≥30)	1,539 (46.7)	79 (4.3)	0.14
Arthritis	851 (24.7)	69 (7.0)	<0.001
Asthma	165 (4.9)	21 (14.6)	<0.001
Sleep problem (nocturnal)	249 (7.4)	55 (22.8)	<0.001
Daily tobacco use	810 (20.4)	46 (4.6)	0.84
Alcohol use (10 drinks or more a week)	158 (3.7)	12 (3.5)	0.68
Insufficient fruits and vegetables	2, 817 (67.7)	124 (4.3)	0.68
Physical inactivity	2,455 (60.5)	100 (4.1)	0.17
Functional disability (WHODAS; 0–100); M (SD)	20.8 (20.1)	33.2 (20.0)	
Low	1,381 (34.5)	24 (1.0)	<0.001
Medium	1,142 (28.3)	54 (3.5)	
High	1,317 (37.2)	82 (7.0)	
Cognitive impairment	263 (8.3)	22 (7.0)	0.15
Verbal learning impaired	1,285 (32.8)	73 (4.9)	0.61
Verbal recall impaired	1,060 (27.2)	53 (4.6)	0.18
Word fluency impaired	1,283 (35.3)	45 (3.9)	0.25
Social cohesion index (range 9–72); M (SD)	22.1 (6.5)	22.9 (6.5)	0.29
Quality of life (QoL) (range 0–100); M (SD)	47.1 (12.5)	37.8 (13.1)	
Low	956 (28.5)	78 (8.1)	<0.001
Medium	1,384 (33.5)	44 (2.6)	
High	1,500 (38.1)	38 (2.0)	

* Chi-square *p*.

### Associations with depression prevalence

In univariate analysis functional disability, low quality of life and chronic conditions (stroke, angina, asthma, arthritis, and nocturnal sleep problems) were associated with past 12 months depression. None of the identified socio-demographic variables, lifestyle factors, cognitive variables, social cohesion, and chronic conditions (high blood pressure, diabetes, obesity) were found to be associated with depression. In multivariable analysis, functional disability, lack of quality of life, and chronic conditions (angina, asthma, arthritis, and nocturnal sleep problems) remained associated with the 12 month depression. Older adults with depression were 7.6 times (95% CI = 3.57–16.35) more likely to have nocturnal sleep problems compared to those without depression, and those with a history of angina were 5.4 times (95% CI = 1.93–10.66) more likely and those with medium functional disability were three times (96% CI = 1.58–5.66) more likely to have depression than those without (see Table 2).

**Table 2:** Multivariable logistic regression with depression in the past 12 months in older South Africans

Sociodemographics	UOR (95% CI)	AOR (% CI)
Gender		
Female	1.00	–
Male	0.91 (0.55–1.53)	
Age		
50–59	1.00	–
60–69	0.86 (0.45–1.64)	
70–79	0.72 (0.21–2.51)	
80 and above	0.42 (0.09–1.80)	
Population group		
Black African	1.00	–
White	0.72 (0.30–1.77)	
Coloured	0.63 (0.33–1.21)	
Indian or Asian	1.02 (0.37–2.75)	
Marital status		
Single	1.00	–
Married	1.12 (0.59–2.11)	
Separated/divorced	1.40 (0.46–4.26)	
Widow	0.97 (0.36–2.66)	
Educational level		
No schooling	1.00	–
Less than primary	1.40 (0.71–2.77)	
Primary	1.40 (0.66–3.12)	
Secondary or more	1.24 (0.57–2.69)	
Wealth		
Low	1.00	–
Medium	1.06 (0.55–2.04)	
High	1.25 (0.80–1.95)	
Geolocality		
Rural	1.00	–
Urban	0.81 (0.40–1.65)	
Health variables		
Chronic conditions		
High blood pressure	0.73 (0.36–1.48)	–
Stroke	3.29 (1.33–8.13)*	1.63 (0.57–4.66)
Angina	5.51 (2.76–11.02)***	4.53 (1.93–10.66)***
Diabetes	1.58 (0.80–3.13)	–
Obesity	1.09 (0.76–1.55)	–
Arthritis	2.29 (1.43–3.67)***	1.68 (1.03–2.66)*
Asthma	4.63 (2.26–9.48)***	3.89 (1.54–9.83)**
Sleep problem (nocturnal)	11.32 (6.57–19.50)***	7.64 (3.57–16.35)***
Functional disability (WHO-DAS)		
Low	1.00	1.00
Medium	3.43 (1.89–6.25)***	2.99 (1.58–5.66)***
High	7.23 (3.49–14.98)***	2.73 (1.09–6.86)*
Daily tobacco use	1.18 (0.44–3.13)	–
Alcohol use (10 drinks or more a week)	0.84 (0.42–1.70)	–
Physical inactivity	1.13 (0.65–1.99)	–
Insufficient fruits and vegetable consumption	1.32 (0.71–2.46)	–
Cognitive impairment	1.93 (0.72–5.15)	–
Verbal learning impaired	1.34 (0.70–2.58)	–
Verbal recall impaired	1.17 (0.88–1.56)	–
Word fluency impaired	0.94 (0.53–1.66)	–
Social cohesion index	1.02 (0.99–1.04)	–
Quality of life		
Low	1.00	1.00
Medium	0.30 (0.18–0.51)***	0.56 (0.33–0.97)*
High	0.23 (0.14–0.39)***	0.68 (0.44–1.07)

* *p*<0.5

** *p*<0.01

*** *p*<0.001.

## Discussion

The study reports for the first time the prevalence of 4.0% of past 12 months depression in a national sample of older adults in South Africa. This prevalence seems to be lower or similar to previous surveys (1, 5–8) and clinically significant depressive symptoms of community-dwelling older adults (2, 9–13). It was also found that depression seemed to be less prevalent among older adults living in communities than among younger community residents (4, 15).

The study found in agreement with previous studies that functional disability (8, 10, 12, 15), low quality of life (8, 11), and chronic conditions (angina, asthma, arthritis, and nocturnal sleep problems) (9, 12, 26, 28–30) were associated with depression. The observation that the mean functional disability of 33.2 in older South Africans with depression compared to those without depression (20.3) confirms the association of functional disability and depression in older adults. Previous research also showed that depressed respondents were not reporting things more negatively for the same level of health or functional disability, and further supports the absence of biased reporting due to depression (48). The data show that comorbidity between chronic physical conditions (angina, asthma, arthritis) and depression is common, and that people with chronic diseases (angina, arthritis, asthma, diabetes) are significantly more likely to suffer from depression than those without, as found in a large study among the general population (48). The risk factors of sleep disturbance and disability are potentially modifiable and their modification could be expected to have an important public health impact (29). As people age, the increase in the incidence of chronic diseases, stressful and emotional events, and the consumption of medications have a substantial impact on sleep quality (49).

Unlike in other studies (7–10, 14, 15, 20, 21), this study did not find any significant socio-economic (gender, population group, socio-economic status, geolocality), behaviour (dietary habits, physical activity), and cognitive impairment (9, 12, 32, 33) differences in relation to depression prevalence. Overall, disease status had a greater association with depression than did socio-demographic characteristics, which conforms with a large multi-country study in the general population (48). However, in a meta-analysis it was also found that some socio-economic factors (higher age, lower education level, being unmarried) did not increase the risk of depression, and there was heterogeneity in the results for the risk factor cognitive impairment (29). Some of these differences may perhaps be related to different definitions of these variables in different studies or due to different study samples (29). For example, studies finding an association between depression and cognitive impairment can be based on primary-care rather than population or community-based samples (50). In the case of primary-care samples, a selection bias occurs regarding help-seeking on the basis of distress or impairment (51). It is possible that we did not find any gender differences because women in this study did not have greater vulnerability to depression due to lower socio-economic and other risk factors such as bereavement and depression history not assessed in this study.

In many primary care settings, patients presenting with multiple disorders that include depression often do not get diagnosed, and if they do, treatment is often focused towards other chronic diseases (48, 52). Depression can be treated in primary care or community settings with locally available cost-effective interventions (48, 53). Reynolds et al. (53) note that ‘additional research is needed to address the specific issues of depression prevention in older adults in low- and middle-income countries (LMICs). The growing number of older adults globally, as well as workforce issues and the expense of interventions, makes it important to develop rational, targeted, and cost-effective risk-reduction strategies. In our opinion, one strategy to address these issues entails the use of lay health counsellors (LHCs), a form of task shifting already shown to be effective in the treatment of common mental disorders in LMICs’ (54, 55). The role of the LHC in the integration of the management of common mental disorders in primary care has been described in detail by Pereira et al. (55), in particular in terms of being a case manager, providing psychoeducation and interpersonal therapy for patients with moderate/severe depression (56). In addition, it is suggested to address other chronic conditions by creating awareness, education regarding healthy behaviours, such as the benefits of being physically active and eating healthy (57).

## Limitations of the study

This study had several limitations. First, the self-report of health variables such as depression symptoms, tobacco or alcohol use should be interpreted with caution; it is possible that measurement errors occurred. In addition, recall bias could have occurred since asking about depressive symptoms may carry social stigma (58), and survival bias may have reduced the prevalence of depression, as depression is associated with mortality (59). Second, information on social stressful life events, such as bereavement, including socio-economic stress factors (12, 18, 19) was not collected and should be included in future studies. Third, this study was based on data collected in a cross-sectional survey. We cannot, therefore, ascribe causality to any of the associated factors in the study. Finally, data were collected from older adults who were available in the household on the day of the survey. Respondents who were institutionalised (prison, hospital, care home) and not returning to the household within 7 days and those who had moved more than 50 km away from the study household were not included, and thus we may have underestimated the prevalence of depression. Probability sampling was used to maximise external validity or generalisability of the results of the study. The Census Bureau found an overall match between the distributions of age, sex, and urban/rural residence in SAGE data, including South Africa, and data from the Census Bureau, United Nations population data, and Central Intelligence Agency's data.

## Conclusion

Self-reported depression in older South Africans seems a public health problem calling for appropriate interventions to reduce the occurrence. Factors identified to be associated with depression, including functional disability, lack of quality of life, and chronic conditions (angina, asthma, arthritis, and nocturnal sleep problems), can be used to guide interventions. The identified protective and risk factors can help in formulating public health care policies to improve quality of life among older adults.
